# Health behavioral responses to parental myocardial infarction and impact on own risk of disease in the general population

**DOI:** 10.3389/fpubh.2023.1200593

**Published:** 2023-07-06

**Authors:** Christian Skouenborg, Martin Lucas Jørgensen, Torben Heien Nielsen, Marianne Benn

**Affiliations:** ^1^Department of Clinical Biochemistry, Copenhagen University Hospital - Rigshospitalet, Copenhagen, Denmark; ^2^Department of Economics, University of Copenhagen, Copenhagen, Denmark; ^3^Center for Economic Behavior and Inequality, University of Copenhagen, Copenhagen, Denmark; ^4^The Copenhagen City Heart Study, Copenhagen University Hospital - Bispebjerg and Frederiksberg Hospital, Copenhagen, Denmark; ^5^Department of Clinical Medicine, Faculty of Health and Medical Sciences, University of Copenhagen, Copenhagen, Denmark

**Keywords:** health behavior, parental disease, myocardial infarction, ischaemic heart disease, 10-years risk score risk

## Abstract

**Aims:**

A family history of coronary heart disease increases one's own risk of experiencing cardiovascular disease and death. An implication of the hereditary nature of the disease is that individuals are provided information about their own risk when a parent is affected, potentially leading them to engage in behaviors that reduce their own risk. In this study, we assessed how a 10-year risk of a cardiovascular event, measured by SCORE, changes for the offspring in response to a parent experiencing a myocardial infarction.

**Methods:**

We analyzed 19,995 individuals from the general population in the Copenhagen City Heart Study of whom 2,071 had a parent, who suffered from a myocardial infarction during four decades of observation using fixed-effects regressions.

**Results:**

Following a parental myocardial infarction, individuals reduced their 10-year risk by 0.16 percentage points constituting a 7.1% reduction of baseline risk. Male participants had the largest change in the risk SCORE following an event of the mother, with a 12.4% reduction from the baseline risk. The degree of response contingent on their own level of risk was found to be the largest for individuals with a 10-year risk between 5% and 10%, who also showed a reduction in systolic blood pressure following paternal myocardial infarction. Parental myocardial infarction was associated with an increased smoking rate in individuals with a baseline risk above 10%, while reductions in risk were seen for individuals with a lower baseline risk.

**Conclusion:**

Following a parental event, individuals reduced their 10-year risk with the largest reductions in their own risk, as observed in men and individuals experiencing a maternal event. The response was the largest for individuals with a 10-year risk for myocardial infarction between 5 and 10%.

## Introduction

It has been established that a family history of coronary heart disease increases one's own risk of experiencing angina pectoris, myocardial infarction, and cardiovascular death (1–5). While this relationship may be attributed to both genetic factors (6–10) and inter-generational transmission of lifestyle factors, such as smoking (11–13), it is of relevance to investigate how cardiovascular risk changes for individuals in the aftermath of a parent experiencing a cardiovascular event. An implication of the hereditary nature of the cardiovascular disease is that individuals are implicitly provided with information about their own personal health and risk when a parent is affected, potentially leading them to engage in behaviors that reduce their own level of risk. Identifying whether individuals respond to this information is of relevance for physicians when attending to the parent as this provides an opportunity to motivate the children of the patient into taking precautionary actions for themselves.

A potential way to establish causality on how risk changes in the aftermath of a parental event is to assess the cardiovascular risk before and after the occurrence of the event. The magnitude of response to the event can then be identified by comparison to individuals who are observed over the same course of time but who do not experience a parental event.

In this study, we first calculated a 10-year risk of fatal cardiovascular disease using the European Society of Cardiology risk SCORE across time for participants in the five repeated examinations of the Copenhagen City Heart Study (1976 through 2018) (14). Second, we tested SCORE by estimating the association between categories of 10-year risk of fatal cardiovascular disease and the risk of ischaemic heart disease, myocardial infarction, and cardiovascular mortality. Finally, we estimated the prospective change in the 10-year risk of fatal cardiovascular disease as a change in SCORE following myocardial infarction in a parent relative to individuals where an event did not occur. It was also investigated whether the magnitude of response, as a change in SCORE, varied contingent on the personal characteristics of the participants and whether the underlying mediator of the change in SCORE could be identified.

## Methods

### The study population

This study used data from a prospective Danish cohort study, the Copenhagen City Heart Study. Participants invited to the study were aged ≥20 years and were randomly selected from the national Danish Civil Registration System to represent the age and sex distribution in the general population. The Copenhagen City Heart Study was initiated in 1976–1978 (1st examination), with follow-up examinations in 1981–1983 (2nd examination), 1991–1994 (3rd examination), 2001–2003 (4th examination), and 2011–2015 (5th examination), each of which invited new participants to further supplement the cohort. The study obtained data from self-administered questionnaires, reviewed by an examiner, physical examinations, and blood samples. Follow-up time began at the first inclusion into the study and ended with censoring at the date of death, emigration (*n* = 868), or end of follow-up on 13 December 2018, whichever came first. Details have been described elsewhere (15).

### Ethics approval

The Committee on Biomedical Research Ethics for the Capital Region in Denmark (H-KF-01-144/01) approved the study. All participants gave written informed consent.

### Parental myocardial infarction

The self-registered questionnaire provided baseline and follow-up information from each examination an individual participated in. Participants were asked that “Have your mother had a myocardial infarction?” (no/yes), and a similar question was asked about their father.

### SCORE in participant

Participants were categorized into categories of 0–4.9%, 5–9.9%, and ≥10% 10-year risk of fatal cardiovascular disease using the European SCORE for a population at low risk of cardiovascular disease (14). This uses information on sex (women/men), smoking status (non-smoker/smoker), age (40–49.9, 50–54.9, 55–59.9, 60–64.9, and ≥65 years), total cholesterol (< 4, 4–4.9, 5–5.9, 6–6.9, 7–7.9, and ≥8 mmol/L), and systolic blood pressure (120–139, 140–159, 160–179, and ≥180 mmHg). As this information was acquired for each participant at every follow-up examination, the algorithm developed by Conroy et al. was used to calculate exact SCORE values for each participant at every follow-up (14). Thus, at each follow-up examination, a participant has a certain combination of relevant covariates which constitute a corresponding 10-year risk SCORE, meaning that changes to SCORE may be due to a number of different factors occurring simultaneously.

### Endpoints in study participants

Diagnoses of ischaemic heart disease and myocardial infarction (WHO ICD 8th edition codes: 410–414; and 10th edition codes: I20-I25) were collected by reviewing all hospital admissions and diagnoses entered in the National Danish Patient Registry and all causes of death entered in the National Danish Causes of Death Registry (16).

### Covariates in study participants

The self-registered questionnaire provided baseline and follow-up information from each examination on smoking status (divided into non-smoker/smoker) and menopause (yes/no). At the physical examinations, systolic/diastolic blood pressure (mmHg) was measured. Hypertension was systolic blood pressure ≥140 mmHg (≥135 mmHg for individuals with diabetes), diastolic blood pressure ≥90 mmHg (≥85 mmHg for individuals with diabetes), or the use of antihypertensive medication prescribed specifically for hypertension. The use of lipid-lowering medication was self-reported and >97% statins. Civil status was divided into unmarried, married, separated, divorced, or widowed. Biochemical analysis of blood samples provided data on blood concentrations and total cholesterol. Missing data on covariates varied from 0 to 1% for any individual variable. Individuals with missing covariates were excluded from analyses.

### Statistical analyses

We used StataSE 16.1(StataCorp, College Station, TX, USA). Student's *t*-test was used for pairwise comparisons. To test whether categories of 10-year risk of fatal cardiovascular disease estimated by SCORE were associated with ischaemic heart disease, myocardial infarction, and cardiovascular death, we used Fine and Gray regression models with age as the time scale (=age adjustment) taking competing risks of death or death of other causes into account and further adjusted for sex and year of birth. Trends for significance across ordered categories of 10-year risk were tested by using the non-parametric Cuzick's extension of a Wilcoxon rank-sum test.

To examine change in SCORE or own risk factors after myocardial infarction in a parent, fixed-effects regressions were used. Unobserved, time-invariant differences in characteristics are likely to be present between the treatment group (individuals who experienced a parental myocardial infarction) and the control group (individuals who did not experience a parental myocardial infarction). If these unobserved characteristics correlate with the treatment variable, i.e., whether a parental event occurs, the unobserved heterogeneity will bias the estimates. This could be the case if a latent trait, i.e., genetic predisposition, determines the underlying risk for both parent and child simultaneously. However, since participants are observed across time, both pre- and post-occurrence of the event, a fixed-effects model can be employed. The fixed-effect estimator was used at the participant level across observed follow-up periods of the five examinations of the Copenhagen City Heart Study, thus accounting for unobserved differences between the two groups. A time-fixed effect was also included in the regression to account for time trends across follow-up periods. In analyses on SCORE, individuals older than 75 years were excluded, as older age may overestimate the effects on SCORE due to the exponential nature of the measure. Clustered standard errors at the participant level were used to control for residual correlation for the same individuals across follow-up periods. For all fixed-effects regressions, multifactorial adjustment was made for civil status, education level, time trends, age, and SCORE (to account for the non-linear association between age and SCORE). The description of the fixed-effect model used for the construction of results is shown in the Supplementary material.

## Results

The baseline characteristics of individuals from the general population in the Copenhagen City Heart Study by parental myocardial infarction are shown in Table 1. Of individuals participating (*n* = 19,995) in the study, 23% (*n* = 2,071) had a parent who had a myocardial infarction. Individuals with a parent with a myocardial infarction were more often women, were slightly older, had higher total cholesterol, had higher systolic blood pressure, more often had hypertension, had a history of ischaemic heart disease and myocardial infarction, more often had used lipid-lowering medication, and more often had < 8 years schooling compared to individuals without a parent with a myocardial infarction.

**Table 1:** Baseline characteristics of individuals from the Danish general population and as a function of event status of the parent.

	**Myocardial infarction in a parent**			
	**No**	**Yes**	***P*** **-value**	**All**
Number of individuals	17,924 (90 %)	2,071 (10 %)	-	19,995 (100%)
Sex, % women	35%	65%	< 0.001	55%
Age at baseline	48.1 (0.1)	52.1 (0.3)	< 0.001	48.7 (0.1)
SCORE^*^, all	2.27 (0.04)	2.14 (0.12)	0.35	2.26 (0.04)
SCORE^*^, men	3.14 (0.07)	2.92 (0.21)	0.42	3.13 (0.06)
SCORE^*^, women	1.52 (0.04)	1.70 (0.15)	0.20	1.53 (0.04)
Total cholesterol, mmol/L	5.9 (0.01)	6.2 (0.02)	< 0.001	5.9 (0.01)
Systolic blood pressure, mmHg	134.5 (0.2)	138.5 (0.4)	< 0.001	134.8 (0.2)
Body mass index, kg/m^2^	25.1 (0.0)	25.4 (0.1)	0.04	25.1 (0.0)
Hypertension (%)	26%	31%	< 0.001	26%
Smoking, current (%)	58%	62%	0.01	58%
Ischaemic heart disease (%)	36%	43%	< 0.001	37%
Acute myocardial infarction (%)	21%	24%	0.034	21%
Lipid lowering treatment (%)	1.0%	3.0%	< 0.001	1%
Education, < 8 years (%)	37%	48%	< 0.001	37%
Physical activity, hours per week (%)	2.2 (0.01)	2.2 (0.02)	< 0.001	2.19 (0.01)
Postmenopausal, women only (%)	61%	78%	< 0.001	63%

Continuous values are summarized as the mean and standard error of the mean of the first observed value of a given individual. Categorical values are summarized as the number and percent of the total. The baseline indicates the latest observation pre-parental myocardial infarction. The p-values are from Student's t-test for continuous variables and Pearson's chi-square test for categorical variables. ^*^SCORE is the mean 10-year risk of fatal cardiovascular disease and the mean dependent variable considered in the analysis.

### Risk of ischaemic heart disease and mortality

**Figure 1:**
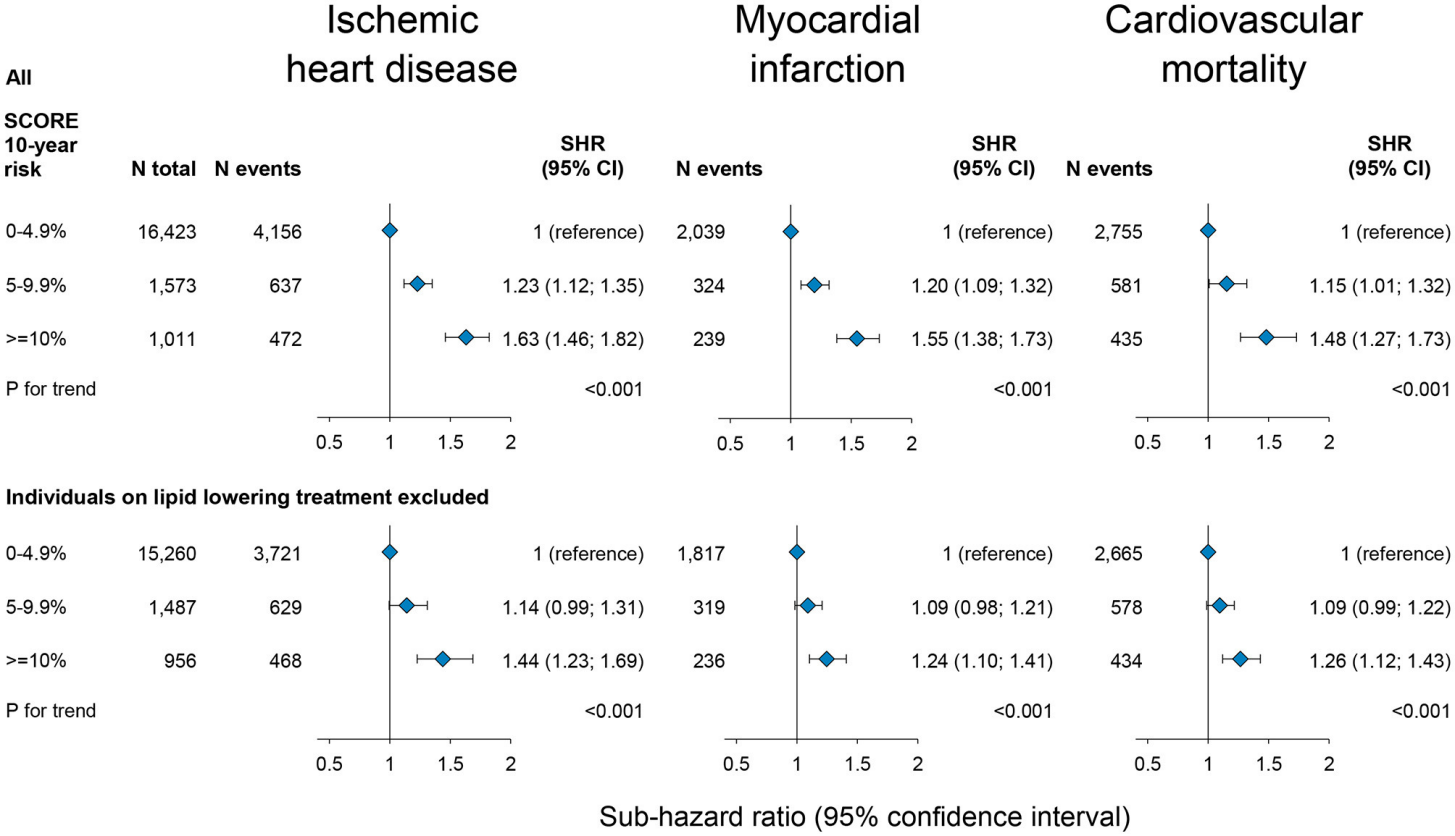
Prospective risk of ischaemic heart disease, myocardial infarction, and cardiovascular mortality as a function of 10-year risk of fatal cardiovascular disease by SCORE in the general population, the Copenhagen City Heart Study. Risk is estimated as sub-hazard ratios (SHR) with 95% confidence intervals (CI) by a Fine and Gray competing risk analysis taking the competing risk of death into account for cardiovascular disease and risk of death of other causes for cardiovascular mortality. Individuals older than 75 years were excluded as older age may overestimate the effects on SCORE due to the exponential nature of the measure. *N* = number.

The prospective risk of ischaemic heart disease, myocardial infarction, and cardiovascular mortality as a function of the 10-year risk of fatal cardiovascular disease categorized using the SCORE is shown in Figure 1. During a median of 24 years of follow-up (range 0–42 years; 430,820 person-years), 5,265 were diagnosed with ischaemic heart disease, 2,602 had a myocardial infarction, and 3,771 died of cardiovascular disease.

As expected, risks of ischaemic heart disease, myocardial infarction, and cardiovascular mortality were stepwise higher with a higher 10-year risk of fatal cardiovascular disease SCORE categories as compared to those with a SCORE of 0–4.9%. For individuals with a SCORE of ≥10% compared to those with a SCORE of 0–4.9%, the age, sex, and birth year adjusted sub-hazard ratios were 1.63 (95% confidence interval: 1.46; 1.82) for ischaemic heart disease, 1.55 (1.38; 1.73) for myocardial infarction, and 1.48 (1.27; 1.73) for cardiovascular mortality (Figure 1). Risks were similar excluding individuals on lipid-lowering treatment (Supplementary Figure 1, lower panel).

### Change in SCORE after myocardial infarction in a parent

**Figure 2:**
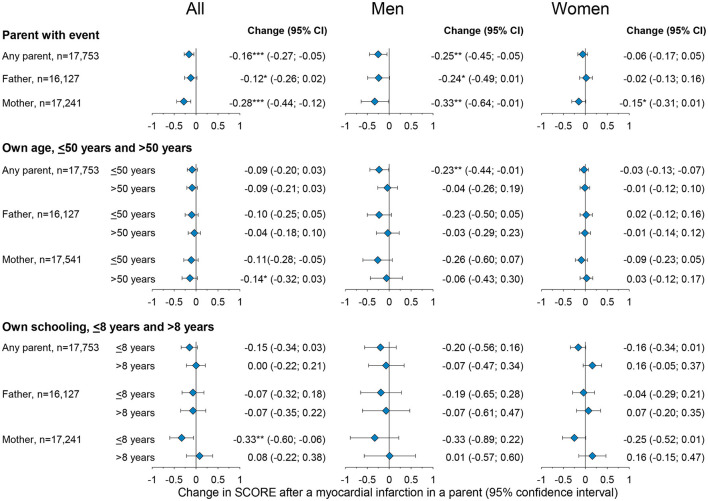
Changes in own 10-year risk of fatal myocardial infarction (SCORE) after a father, a mother, or either parent experience a myocardial infarction in the general population in the Copenhagen City Heart Study. The upper panels show overall effects, the middle panels by own age, and the lower panel by years of schooling. Estimates are by fixed-effects regressions, estimating the effect of paternal, maternal, and parental myocardial infarction on their own SCORE risk. The fixed-effect estimator was at the participant level across observed follow-up periods between the five examinations of the Copenhagen City Heart study. Confidence intervals (CI) are reported in parentheses. *p-value < 0.05, **p-value < 0.01, and ***p-value < 0.001. *N* = number. The full table including covariate estimates can be found in Supplementary Tables 1–3.

Figure 2 shows the effect of a parental myocardial infarction on own cardiovascular risk SCORE dependent on own as well as parental sex by fixed-effects regression. An event in either parent was associated with a 0.16 percentage point reduction in SCORE (−0.27; −0.05), constituting a 7.1% reduction in baseline risk [(0.16/2.26)^*^100 = 7.1%, where 2.26 is the observed baseline SCORE in the population as reported in Table 1] (Figure 2, upper panels; Supplementary Table 1). A maternal myocardial infarction had the largest effect on own 10-year risk of fatal cardiovascular disease with a 0.28 percentage point reduction in SCORE (−0.44; 0.12), constituting a 12.4% reduction of baseline risk, whereas a paternal myocardial infarction associated with a reduction of 0.12 percentage point (−0.26; 0.02), constituting a 5.3% reduction of baseline risk.

The effect of a parental event on own cardiovascular SCORE dependent on own age is shown in Figure 2, middle panel and Supplementary Table 2. Individuals were categorized by age into ≤ 50 years and >50 years, with individuals ≤ 50 years serving as the baseline of the analysis. Effects were estimated by fixed-effects regression using interaction terms for own age being >50 years, parental myocardial infarction, and the marginal effects of these two factors. Being >50 years does not impact the magnitude of SCORE reduction associated with a parental event since neither of the interaction terms provides statistically significant results.

The effect of the duration of schooling in response to a parental event is shown in Figure 2 lower panel and Supplementary Table 3. Individuals were categorized by < 8 years vs. ≥8 years of schooling, with schooling < 8 years serving as the baseline. The results suggest that having ≥8 years of schooling does not impact the magnitude of SCORE reduction associated with a parental event since neither of the interaction terms provides statistically significant results.

### Change in own SCORE after myocardial infarction in a parent—Effect of own baseline risk

**Figure 3:**
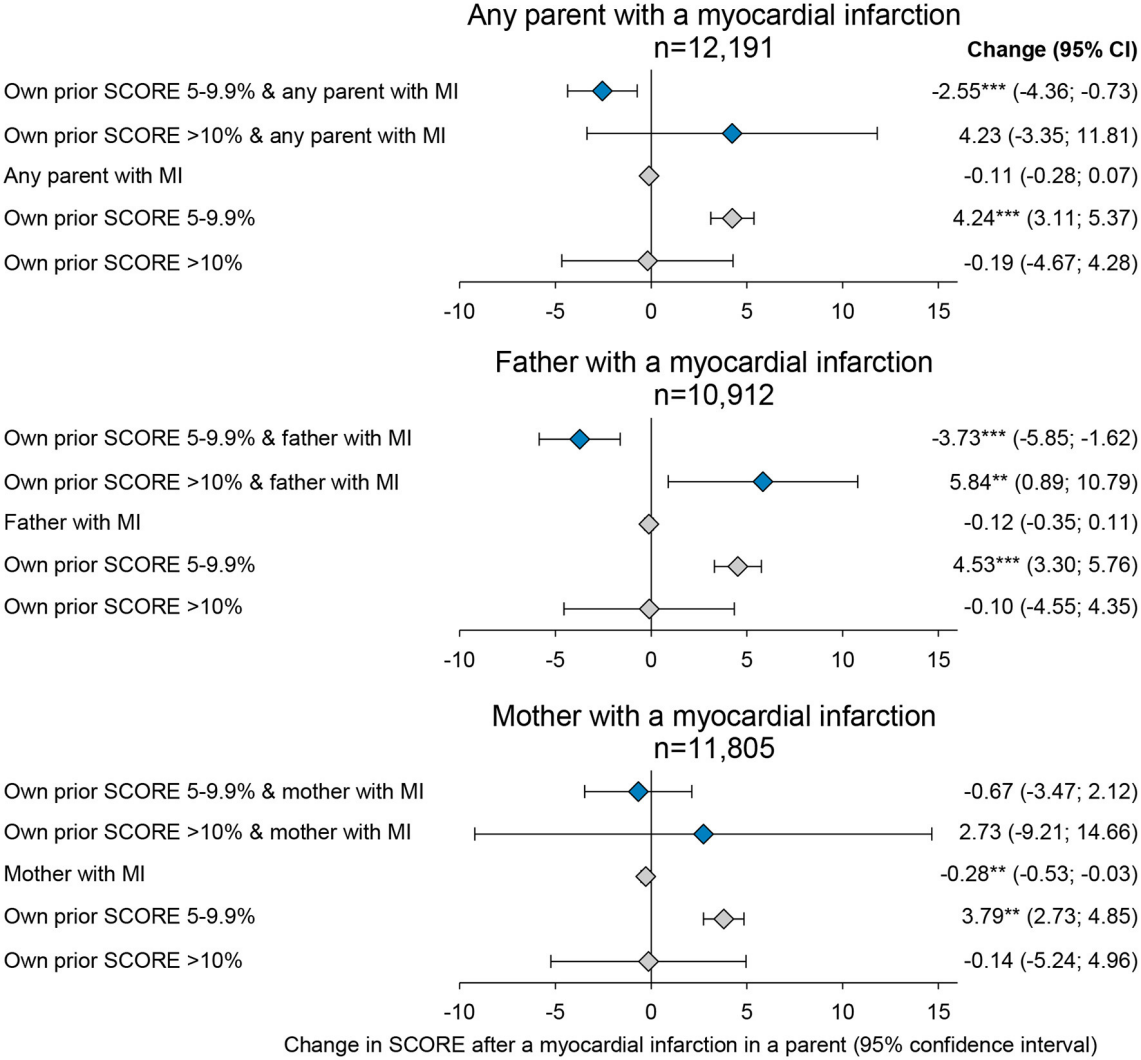
Changes in their own 10-year risk of fatal myocardial infarction (SCORE) after a parent experiences a myocardial infarction by own risk SCORE before the parental event. Estimates are obtained by fixed-effects regressions, estimating the effect of any parent, a father, or a mother experiencing a myocardial infarction on their own SCORE risk. Estimates shown with blue diamonds are the interaction estimates and gray diamonds are the single estimates. The fixed-effect estimator was at the participant level across observed follow-up periods between the five examinations of the Copenhagen City Heart study. Confidence intervals (CI) are reported in parentheses. **p-value < 0.01, and ***p-value < 0.001. *N* = number. The full table including covariate estimates can be found in Supplementary Table 4.

The 10-year risk of fatal cardiovascular disease estimated as participant SCORE values in the previous follow-up period of any given period was used to categorize individuals into a prior SCORE below 5%, 5–10%, and >10% (referred to as baseline risk). Interaction between the effect of a parental myocardial infarction and the baseline risk of the individual was examined using individuals with baseline risk < 5% as a reference and adding binary indicators for the two other SCORE-intervals to the regression, together with interaction terms between these and parental events (Figure 3; Supplementary Table 4).

For individuals with baseline risk >5%, an event in either parent was associated with a 0.11 percentage point reduction in SCORE (−0.28; 0.07). For individuals with baseline risk between 5 and 10%, an event in either parent was associated with an additional 2.55 percentage point reduction in SCORE (−4.63; −0.73) compared to individuals with baseline risk >5%, thus indicating a 26–51% reduction in SCORE. For individuals with baseline risk >10%, an event was associated with a non-statistically significant increase in a SCORE of 4.23 percentage points (−3.35; 11.81).

### Change in SCORE-components after myocardial infarction in a parent

**Figure 4:**
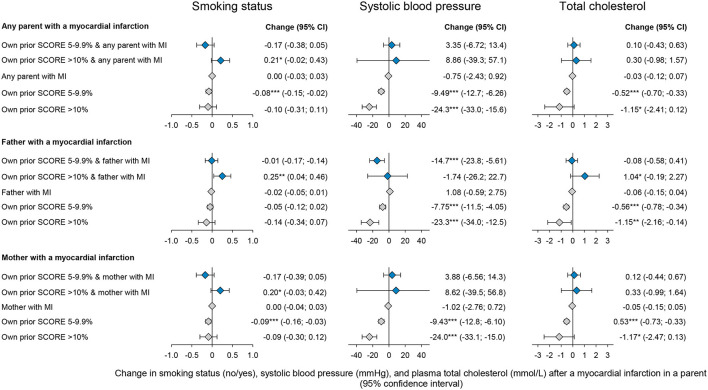
Changes in own cardiovascular risk factors following a myocardial infarct in the father, mother, or either parent by own risk before the parental event. Estimates are by fixed-effects regressions, estimating the effect of any parent, a father, or a mother experiencing a myocardial infarction. Estimates shown with blue diamonds are the interaction estimates and gray diamonds are the single estimates. The fixed-effect estimator was at the participant level across observed follow-up periods between the five examinations of the Copenhagen City Heart study. Confidence intervals (CI) are reported in parentheses. *p-value < 0.05, **p-value < 0.01, and ***p-value < 0.001. *N* = number. The full table including covariate estimates can be found in Supplementary Table 5.

To examine whether the differential effects of baseline risk are driven by a single component of the SCORE, separate regressions of smoking status, systolic blood pressure, and total cholesterol as outcome variables were estimated (Figure 4; Supplementary Table 5).

For individuals with baseline risks >10%, a maternal myocardial infarction was associated with an increased smoking rate of 20 percentage points (−0.03:0,42) and a paternal event with a 25 percentage points increase (0.04:0.46). For individuals with baseline risks of 5–10%, a parental myocardial infarction was associated with smoking cessation; however, estimates were not significant examining an event in a father or a mother separately. Systolic blood pressure declined by 14.69 mmHg (−23.78:-5.62) for individuals with a baseline SCORE of 5–10% and a myocardial infarction in a father. For all other groups, systolic blood pressure did not change. Moreover, total cholesterol did not change across subgroups.

## Discussion

Our results suggest that individuals who experience a parental myocardial infarction responded with a positive change in their own risk of cardiovascular disease, as measured by 10-year risk SCORE. Reductions in SCORE were driven by individuals with a SCORE of 5–10% for a 10-year risk of fatal disease prior to the parental event. The reductions in 10-year risk SCORE could only be attributed to risk-reducing changes in systolic blood pressure in individuals with a SCORE between 5 and 10%. The results showed an increase in smoking cessation in connection with a paternal myocardial infarction. However, the effects varied by individual baseline risk, with individuals with a risk SCORE above 10% increased smoking rates, while individuals with a SCORE of 5–10% showed a decline in smoking rates.

To the best of our knowledge, only two other studies have addressed how the status of health-related behaviors changes among children following a parental health event. Using the original- and offspring cohort of the Framingham heart study, Darden et al. (17) studied changes in smoking behavior and self-reported health status among offspring of individuals suffering from both smoking- and non-smoking-related health events. Their analysis included 2,402 offsprings from 1971 to 2002 with a total of 15,843 follow-up observations. They found no significant reduction in smoking among male participants with a parent experiencing cardiovascular disease. However, the likelihood of smoking cessation was significant for male participants and decreased if the father of the participant was reported to be a non-smoker and experienced a cardiovascular event. For women, a significant reduction in smoking was observed for both paternal and maternal cardiovascular events.

Fadlon et al. (18) investigated how the use of lipid-lowering statins changes in response to health events in people with different familial and social relations to the individual such as spouse, sibling, parent, in-laws, and co-workers. Using Danish administrative data, they applied a difference-in-difference estimation approach different from the one presented in this study. The control group in this study consisted of individuals who did not experience a parental event in the investigated period or had not yet experienced it at a certain point in the time interval analyzed, whereas the control group in Fadlon and Nielsen (18) study consisted of individuals where the parental event had not yet occurred but would at a later time point. Following a parental cardiovascular event, Fadlon and Nielsen (18) found that the use of lipid-lowering statins not only increased relative to the control group but also the gap remained persistent and increasing over time. Their study additionally provided findings relevant to our analysis, that we were not able to investigate within our own data. Particularly, they analyzed the response to the use of lipid-lowering statins following the event of a mother- or father-in-law. The responses are also found to be present following such types of events albeit in lower magnitudes than that for biological parents. This suggests that responses are due to information both concerning biological risk and lifestyle risks.

We found that the results of this study are in line with and support the main findings of Fadlon and Nielsen (18) study concerning parental events and that our analysis provide additional evidence on the matter investigated. First, our study supports that individuals respond to parental events, shown by 10-year risk SCORE which is a clinically recognized measure to predict a 10-year risk of cardiovascular disease. Second, we show that the magnitude of response was highly influenced by one's own health status, given that individuals with a SCORE >10% are less inclined to change their risk following a parental event contrary to individuals with a SCORE between 5 and 10%.

Gender differences and the role of education in response to parental health events have not, to the best of our knowledge, been directly examined in other studies. However, further research studies have been conducted on health disparities between sexes and socioeconomic status. The smaller reduction in 10-year risk SCORE found for women relative to men could be an implication of the misconception within the investigated period that women's risk of cardiovascular disease is lower compared to men (19–21) but may also be a result of lower SCORE levels among women in general relative to men. Although a significant role of education was not found in our analysis, we argue that further research is needed on the matter since several publications have described the disparities in cardiovascular risk implicated by differences in socioeconomic status (22–24).

There are limitations to this study. Individuals included in the study inhabit a limited geographic area and a low-risk, affluent population, which means that the results should primarily be extrapolated to similar low-risk Western populations. It could be argued, however, that the concept of altering risk in the aftermath of a parental event, in general, is applicable to other populations. As smoking is self-reported and used in the calculation of the 10-year risk SCORE, our data may be influenced by social desirability bias, leading to a potential underestimation of SCORE levels in certain cases. Recently, an updated SCORE algorithm has been published, SCORE2 (25), where non-HDL cholesterol is used rather than total cholesterol. However, in this study, we have chosen to analyse our data in alignment with the original 10-year risk SCORE since patients in our study have been assessed for risk by physicians in accordance with the original algorithm. To the best of our knowledge, this is the first study to report data on change in risk due to a parental event and why this study should be replicated before firm recommendations are made.

The finding that individuals react with changing their risk of cardiovascular disease, measured by 10-year risk SCORE in correspondence to myocardial infarction in a parent, and that the responses differ between groups of higher and lower personal risk of cardiovascular disease, suggests that health inequalities are present both before and after a parental event. The results presented in this study, along with the studies by Fadlon and Nielsen (18) and Darden et al. (17) do, however, indicate that a change in health behaviors, such as the use of lipid-lowering treatment and smoking cessation, as well as risk, occur in the aftermath of a parental event. This in turn supports the notion that individuals are in fact motivated to change their lifestyle when confronted with a parent experiencing an event. Hence, relatives should be encouraged to contact their general practitioners with the purpose of being screened and oriented to their own risk of developing cardiovascular disease and how changes in health behaviors can help lower this risk. This intervention may be implemented *via* guidelines and patient consultations with cardiologists or general practitioners where next-of-kin are present but could also be done *via* public health campaigns and evidence-based digital solutions specifically targeting the relatives of patients. Furthermore, the capability of change seems to be greatest in the medium-risk group (SCORE of 5–10%) as compared to high-risk individuals (>10%), who do not display significant changes to their health behaviors after parental myocardial infarction. Thus, specifically targeting high-risk individuals would yield the highest impact of these interventions, which in turn may help mitigate health inequalities and reduce the prevalence of cardiovascular events.

In conclusion, a parental myocardial infarction is associated with changes in risk for the offspring. The magnitude of response differs in the contingency of their own level of risk. Thus, policy recommendations should also target relatives of patients with myocardial infarction.

## Data availability statement

The raw data supporting the conclusions of this article will be made available by the authors, without undue reservation.

## Ethics statement

The studies involving human participants were reviewed and approved by the Committee on Biomedical Research Ethics for the Capital Region in Denmark (H-KF-01-144/01) approved the study. The patients/participants provided their written informed consent to participate in this study.

## Author contributions

CS and MJ wrote the first draft of the manuscript. TN and MB contributed to revisions. All authors contributed to the conceptualization of the project, data curation, formal data analyses, validation, visualization, and had access to all data.
