# Supplementary material for: Health behavioral responses to parental myocardial infarction and impact on own risk of disease in the general population
Source: Front Public Health. 2023 Jul 6;11:1200593. doi: 10.3389/fpubh.2023.1200593 (PMC10359892; doi:10.3389/fpubh.2023.1200593)
Supplement: Supplementary file 1 [file Data_Sheet_1.pdf]

# Health behavioural responses to parental myocardial infarction and impact on own risk of disease in the general population

Christian Skouenborg, MSc Economics*[1], Martin Lucas Jørgensen MSc Economics*[1], Torben Heien Nielsen MSc Economics, PhD[2,5], and Marianne Benn, MD, DMSc, PhD[1,3,4]

[*]These authors contributed equally to this work

[1]Department of Clinical Biochemistry, Copenhagen University Hospital - Rigshospitalet, Denmark;

[2]Department of Economics, University of Copenhagen, Copenhagen, Denmark; [3]The Copenhagen City Heart Study, Copenhagen University Hospital – Bispebjerg and Frederiksberg Hospital, Copenhagen, Denmark; [4]Department of Clinical Medicine, Faculty of Health and Medical Sciences, University of Copenhagen, Copenhagen, Denmark; Centre for Economic Behaviour and Inequality, University of Copenhagen, Copenhagen, Denmark.

## Content

Appendix 1. Statistical method used to construct Figure 2, 3 and 4 ........ 2

Supplementary Figure 1 ........ 2

Supplementary Table 1 – extension of data shown in Figure 2, upper panel........ 7

Supplementary Table 2 – extension of data shown in Figure 2, middle panel........ 9

Supplementary Table 3 – extension of data shown in Figure 2, lower panel........ 12

Supplementary Table 4 – extension of data shown in Figure 3 ........ 15

Supplementary Table 5 – extension of data shown in Figure 4 ........ 17

## Appendix 1. Statistical method used to construct Figure 2, 3 and 4

The appendix provides a description of how we constructed Figures 2-4. The overarching goal was to estimate the effect on 10-year risk SCORE for adult children (subjects in the data) that experienced a parent suffering from a myocardial infarction (MI).

Ideally, we would have had information for the same families in both the hypothetical states where the MI had and had not occurred. That would provide the causal impact of a parental event on SCORE. Naturally, only one of the states are observed in reality – either families experienced an MI, or they did not. Consequently, the statistical analysis compares these two types of families.

Causal interpretation of a simple comparison of SCORE level contingent on whether or not a parental MI occurs, builds on the assumption that the likelihood of a parental event is uncorrelated with the SCORE level of their children. The validity of this assumption is highly disputable if any selection into the event (or residual confounding) is present. We take two means to circumvent this concern in our regression analyses. First, we control for covariates in the data that are observable and likely correlated with both parental and own risk of MI. Second, we exploit the longitudinal features of the data that provides multiple measures at different time points for the same individuals. Particularly, this allows for estimating the regressions using fixed-effect methods. By using STATA's *xtreg* procedures our regressions effectively transform the estimation variables into deviation from the mean for each individual (a within-transformation). This way the analysis controls for any unobserved characteristic within the individual that does not change over time, e.g., characteristics affecting MI that is inherited across generations either biologically or through shared health behaviours.

Practically, we estimate the following regressions (with standard errors clustered at the subject level):

*Equation 1, Figure 2, upper panel:*

$$SCORE_{it} = \beta_1 Parental\_MI_{it} + Covariates'_{kit}\delta_k + CCHS'_t\gamma_t + \alpha_i + \varepsilon_{it}$$

where $SCORE_{it}$ is the 10-year risk of experiencing a myocardial infarction for a given individual *i* at a given follow-up *t*. $Parental\_MI_{it}$ is a binary variable indicating if individual *i* reports that a parent has suffered from a myocardial infarct at a given follow-up. $Covariates'_{kit}$ is a vector of *k* covariates that may influence SCORE. These include age, civic status and if the individual has more than eight years of schooling. $CCHS'_t$ are indicators for each of the CCHS examinations and control for time-dependent effects. $\alpha_i$ are individual time-invariant effects. If these unobserved fixed-effects are correlated with both $Parental\_MI_{it}$ and $SCORE_{it}$, we will get inconsistent and biased estimates

of $\beta_1$. This concern is eliminated by the within-transformation of our data which implicitly controls for these unobserved fixed effects. $\varepsilon_{it}$ is an idiosyncratic term.

*Equation 2, Figure 2, middle panel:*

$$SCORE_{it} = \beta_1 Parental\_MI_{it} + \beta_2 Age\_50_{it} + \beta_3 Age_{50,it} \times Parental\_MI_{it} + Covariates'_{kit}\delta_k + CCHS'_t\gamma_t + \alpha_i + \varepsilon_{it}$$

Equation 2 augments Equation 1 with a binary variable, $Age\_50_{it}$ , indicating whether a subject is 50 years of age or older, and its interaction with $Parental\_MI_{it}$. $\beta_2$ measures the effect of being older than fifty for the SCORE, and $\beta_3$ capture the additional SCORE-effect of having experienced a parental MI for people aged 50 and above.

*Equation 3, Figure 2, lower panel:*

$$SCORE_{it} = \beta_1 Parental\_MI_{it} + \beta_2 Educ_{it} \times Parental_{MI_{it_4}} + Covariates'_{kit}\delta_k + CCHS'_t\gamma_t + \alpha_i + \varepsilon_{it}$$

Equation 3 augments Equation 1 with a interaction between the indicator of whether or not the subject has obtained more than eight years of schooling, and $Parental\_MI_{it}$. Hence, $\beta_2$ captures the additional SCORE-effect of having experienced a parental MI for people with more than eight years of schooling.

*Equation 4, Figure 3:*

$$SCORE_{it} = \beta_1 Parental\_MI_{it} + \beta_2 R2_{it-1} + \beta_3 R3_{it-1} + \beta_4 R2_{it-1} \times Parental\_MI_{it} + \beta_5 R3_{it-1} \times Parental\_MI_{it} + Covariates'_{kit}\delta_k + CCHS'_t\gamma_t + \alpha_i + \varepsilon_{it}$$

Equation 4 augments Equation 1 with the binary variables $R2_{it-1}$, indicating if the 10-year risk score of an individual was between 5% and 10% in follow-up t-1 (at the examination prior to the outcome measurement), and $R3_{it-1}$, indicating if the 10-year risk score of an individual was >10% in follow-up t-1. Furthermore, the regression includes these variables' interaction with $Parental\_MI_{it}$.

*Equation 5, Figure 4:*

$Risk\ Factor_{it} = \beta_1 Parental_MI_{it} + \beta_2 R2_{it-1} + \beta_3 R3_{it-1} + \beta_4 R2_{it-1} \times Parental_MI_{it} + \beta_5 R3_{it-1} \times Parental_MI_{it} + Covariates'_{kit}\delta_k + CCHS'_t\gamma_t + \alpha_i + \varepsilon_{it}$Where $Risk\ Factor_{it}$ is a given risk factor associated with increasing the risk of experiencing a myocardial infarction for a given individual *I* at a given follow-up *t*. The binary variables $R2_{it-1}$, indicates if the 10-year risk

score of an individual was between 5% and 10% in follow-up t-1 (at the examination prior to the outcome measurement), and $R3_{it-1}$, indicates if the 10-year risk score of an individual was >10% in follow-up t-1. Furthermore, the regression includes these variables' interaction with $Parental\_MI_{it}$. See *Equation 1* for a description of the remaining variables.

## Supplementary Figure 1

**All**

| SCORE 10-year risk | N total | Ischemic heart disease: N events | Ischemic heart disease: SHR (95% CI) | Myocardial infarction: N events | Myocardial infarction: SHR (95% CI) | Cardiovascular mortality: N events | Cardiovascular mortality: SHR (95% CI) |
|---|---|---|---|---|---|---|---|
| 0-4.9% | 16,423 | 4,156 | 1 (reference) | 2,039 | 1 (reference) | 2,755 | 1 (reference) |
| 5-9.9% | 1,573 | 637 | 1.23 (1.12; 1.35) | 324 | 1.20 (1.09; 1.32) | 581 | 1.15 (1.01; 1.32) |
| >=10% | 1,011 | 472 | 1.63 (1.46; 1.82) | 239 | 1.55 (1.38; 1.73) | 435 | 1.48 (1.27; 1.73) |
| P for trend | | | <0.001 | | <0.001 | | <0.001 |

**Individuals on lipid lowering treatment excluded**

| SCORE 10-year risk | N total | Ischemic heart disease: N events | Ischemic heart disease: SHR (95% CI) | Myocardial infarction: N events | Myocardial infarction: SHR (95% CI) | Cardiovascular mortality: N events | Cardiovascular mortality: SHR (95% CI) |
|---|---|---|---|---|---|---|---|
| 0-4.9% | 15,260 | 3,721 | 1 (reference) | 1,817 | 1 (reference) | 2,665 | 1 (reference) |
| 5-9.9% | 1,487 | 629 | 1.14 (0.99; 1.31) | 319 | 1.09 (0.98; 1.21) | 578 | 1.09 (0.99; 1.22) |
| >=10% | 956 | 468 | 1.44 (1.23; 1.69) | 236 | 1.24 (1.10; 1.41) | 434 | 1.26 (1.12; 1.43) |
| P for trend | | | <0.001 | | <0.001 | | <0.001 |

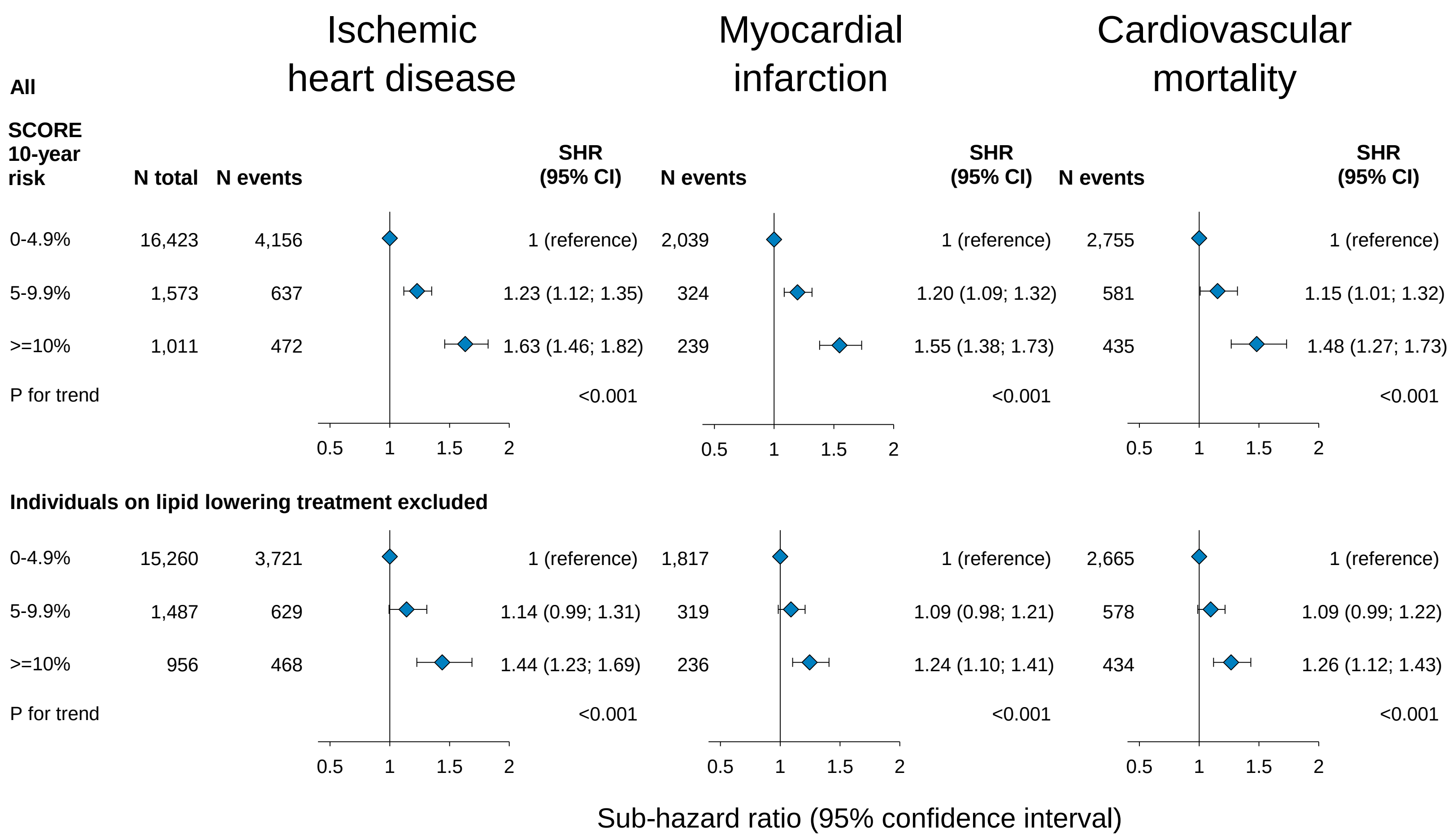


Prospective risk of ischaemic heart disease, myocardial infarction, and cardiovascular disease and cardiovascular mortality as a function of 10-year risk of fatal cardiovascular disease by SCORE in the general population, the Copenhagen City Heart Study. Risk is estimates as sub-hazard ratios (HR) with 95% confidence intervals (CI) by a Fine and Grey competing risk analysis taking competing risk of death into account for cardiovascular

disease and risk of death of other causes for cardiovascular mortality. Individuals older than 75 years were excluded, as older age may overestimate the effects on SCORE due to the exponential nature of the measure. N=number.

## Supplementary Table 1 – extension of data shown in Figure 2, upper panel

Change in risk SCORE following a myocardial infarction of the participants father, mother or either parent

| | All | | | Men | | | Women | | |
|---|---|---|---|---|---|---|---|---|---|
| | Father | Mother | Parent | Father | Mother | Parent | Father | Mother | Parent |
| Parent with MI | | | -0.16*** | | | -0.25** | | | -0.06 |
| | | | (-0.27; -0.05) | | | (-0.45; -0.05) | | | (-0.17; 0.05) |
| Father with MI | -0.12* | | | -0.24* | | | 0.02 | | |
| | (-0.26; 0.02) | | | (-0.49; 0.01) | | | (-0.13; 0.16) | | |
| Mother with MI | | -0.28*** | | | -0.33** | | | -0.15* | |
| | | (-0.44; -0.12) | | | (-0.64; -0.01) | | | (-0.31; 0.01) | |
| Age, years | -0.58*** | -0.54*** | -0.56*** | -0.60*** | -0.56*** | -0.57*** | -0.53*** | -0.52*** | -0.54*** |
| | (-0.66; -0.49) | (-0.63; -0.46) | (-0.64; -0.48) | (-0.74; -0.46) | (-0.70; -0.43) | (-0.70; -0.44) | (-0.63; -0.43) | (-0.61; -0.42) | (-0.63; -0.45) |
| Age, squared | 0.01*** | 0.01*** | 0.01*** | 0.01*** | 0.01*** | 0.01*** | 0.01*** | 0.01*** | 0.01*** |
| | (0.01; 0.01) | (0.01; 0.01) | (0.01; 0.01) | (0.01; 0.01) | (0.01; 0.01) | (0.01; 0.01) | (0.01; 0.01) | (0.01; 0.01) | (0.01; 0.01) |
| Unmarried | -0.19*** | -0.13* | -0.15** | -0.19 | -0.05 | -0.12 | -0.43*** | -0.42*** | -0.41*** |
| | (-0.33; -0.05) | (-0.26; 0.00) | (-0.28; -0.03) | (-0.45; 0.08) | (-0.29; 0.18) | (-0.35; 0.11) | (-0.59; -0.27) | (-0.56; -0.27) | (-0.55; -0.27) |
| Separated | -0.68*** | -0.54*** | -0.57*** | -0.56*** | -0.33** | -0.40** | -0.89*** | -0.85*** | -0.84*** |
| | (-0.88; -0.47) | (-0.73; -0.36) | (-0.75; -0.38) | (-0.93; -0.19) | (-0.66; -0.00) | (-0.73; -0.07) | (-1.13; -0.65) | (-1.07; -0.63) | (-1.05; -0.62) |
| Divorced | -0.30*** | -0.25*** | -0.25*** | -0.18 | -0.09 | -0.11 | -0.48*** | -0.44*** | -0.44*** |
| | (-0.48; -0.11) | (-0.42; -0.08) | (-0.42; -0.08) | (-0.52; 0.16) | (-0.41; 0.23) | (-0.41; 0.19) | (-0.68; -0.29) | (-0.62; -0.25) | (-0.62; -0.26) |
| Widowed | -0.41*** | -0.29** | -0.32*** | -0.15 | 0.24 | 0.05 | -0.20 | -0.18 | -0.16 |
| | (-0.66; -0.17) | (-0.52; -0.07) | (-0.54; -0.10) | (-0.79; 0.50) | (-0.36; 0.84) | (-0.51; 0.61) | (-0.46; 0.05) | (-0.41; 0.06) | (-0.39; 0.07) |
| Schooling >8 years | -0.04 | -0.01 | -0.03 | -0.18 | -0.07 | -0.10 | 0.18 | 0.14 | 0.13 |
| | (-0.23; 0.15) | (-0.19; 0.16) | (-0.19; 0.14) | (-0.48; 0.12) | (-0.34; 0.20) | (-0.35; 0.15) | (-0.04; 0.41) | (-0.09; 0.36) | (-0.09; 0.34) |
| CCHS #2 | 0.27 | 0.06 | 0.13 | 0.24 | -0.05 | 0.01 | 0.25 | 0.20 | 0.25 |
| | (-0.18; 0.73) | (-0.37; 0.50) | (-0.30; 0.55) | (-0.52; 0.99) | (-0.75; 0.65) | (-0.69; 0.71) | (-0.27; 0.78) | (-0.31; 0.71) | (-0.25; 0.74) |

| | | | | | | | | | |
|---|---|---|---|---|---|---|---|---|---|
| CCHS #3 | 0.56 | 0.02 | 0.16 | 0.44 | -0.27 | -0.17 | 0.24 | 0.11 | 0.23 |
| | (-0.79; 1.91) | (-1.28; 1.31) | (-1.11; 1.43) | (-1.75; 2.64) | (-2.37; 1.82) | (-2.24; 1.89) | (-1.33; 1.80) | (-1.41; 1.64) | (-1.24; 1.70) |
| CCHS #4 | -0.06 | -0.93 | -0.70 | -0.60 | -1.65 | -1.53 | -0.40 | -0.67 | -0.46 |
| | (-2.20; 2.09) | (-2.98; 1.13) | (-2.71; 1.31) | (-4.09; 2.90) | (-4.98; 1.69) | (-4.81; 1.75) | (-2.88; 2.08) | (-3.08; 1.74) | (-2.79; 1.87) |
| CCHS #5 | -0.33 | -1.52 | -1.22 | -1.04 | -2.46 | -2.35 | -0.96 | -1.33 | -1.02 |
| | (-3.42; 2.76) | (-4.49; 1.45) | (-4.12; 1.68) | (-6.07; 3.98) | (-7.26; 2.34) | (-7.06; 2.37) | (-4.53; 2.61) | (-4.80; 2.14) | (-4.38; 2.33) |
| Constant | 10.88*** | 9.54*** | 10.09*** | 10.70*** | 9.11*** | 9.37*** | 9.56*** | 9.20*** | 9.85*** |
| | (7.26; 14.50) | (6.06; 13.02) | (6.67; 13.51) | (4.86; 16.54) | (3.51; 14.71) | (3.83; 14.90) | (5.37; 13.76) | (5.13; 13.27) | (5.90; 13.80) |
| Observations | 33,144 | 36,314 | 38,252 | 14,752 | 16,022 | 17,008 | 18,392 | 20,292 | 21,244 |
| R-squared | 0.56 | 0.57 | 0.57 | 0.59 | 0.59 | 0.59 | 0.57 | 0.58 | 0.58 |
| N | 16,127 | 17,241 | 17,753 | 7,405 | 7,863 | 8,160 | 8,722 | 9,378 | 9,593 |

Change in SCORE after a myocardial infarction in a parent in the general population, the Copenhagen City Heart Study, using data from the 2$^{nd}$(#2), 3$^{rd}$(#3), 4$^{th}$(#4), and 5$^{th}$(#5) examinations. Estimates are by fixed-effects regressions, estimating the effect of paternal, maternal, and parental myocardial infarction on own SCORE risk by sex. The fixed-effect estimator was at participant level across observed follow-up periods between the five examinations of the Copenhagen City Heart study. Individuals older than 75 years were excluded, as older age may overestimate the effects on SCORE due to the exponential nature of the measure. Robust confidence intervals are reported in parenthesis. *p value<0.05, **p value<0.01, and ***p value<0.001. N=number, MI=myocardial infarction, CCHS=Copenhagen City Heart Study.

## Supplementary Table 2 – extension of data shown in Figure 2, middle panel

Interactional effects on change in SCORE between own age above 50 years and the mother, father or either parent experiencing a myocardial infarction

| | All | | | Men | | | Women | | |
|---|---|---|---|---|---|---|---|---|---|
| | Father | Mother | Parent | Father | Mother | Parent | Father | Mother | Parent |
| Age>50yr * Parent with MI | | | -0.09 | | | -0.04 | | | -0.01 |
| | | | (-0.21; 0.03) | | | (-0.26; 0.19) | | | (-0.12; 0.10) |
| Age >50yr * father with MI | -0.04 | | | -0.03 | | | -0.01 | | |
| | (-0.18; 0.10) | | | (-0.29; 0.23) | | | (-0.14; 0.12) | | |
| Age>50yr * Mother with MI | | -0.14* | | | -0.06 | | | 0.03 | |
| | | (-0.32; 0.03) | | | (-0.43; 0.30) | | | (-0.12; 0.17) | |
| Parent with MI | | | -0.09 | | | -0.23** | | | -0.03 |
| | | | (-0.20; 0.03) | | | (-0.44; -0.01) | | | (-0.13; 0.07) |
| Father with MI | -0.10 | | | -0.23 | | | 0.02 | | |
| | (-0.25; 0.05) | | | (-0.50; 0.05) | | | (-0.12; 0.16) | | |
| Mother with MI | | -0.11 | | | -0.26 | | | -0.09 | |
| | | (-0.28; 0.05) | | | (-0.60; 0.07) | | | (-0.23; 0.05) | |
| Age >50 years | -1.31*** | -1.32*** | -1.30*** | -1.29*** | -1.31*** | -1.29*** | -1.38*** | -1.39*** | -1.39*** |
| | (-1.40; -1.22) | (-1.40; -1.24) | (-1.38; -1.22) | (-1.44; -1.13) | (-1.45; -1.18) | (-1.43; -1.15) | (-1.47; -1.29) | (-1.48; -1.31) | (-1.48; -1.30) |
| Age, years | -0.52*** | -0.49*** | -0.50*** | -0.55*** | -0.51*** | -0.51*** | -0.47*** | -0.46*** | -0.48*** |
| | (-0.61; -0.44) | (-0.57; -0.41) | (-0.58; -0.43) | (-0.69; -0.42) | (-0.64; -0.38) | (-0.64; -0.39) | (-0.56; -0.37) | (-0.55; -0.37) | (-0.57; -0.39) |
| Age, squared | 0.01*** | 0.01*** | 0.01*** | 0.01*** | 0.01*** | 0.01*** | 0.01*** | 0.01*** | 0.01*** |
| | (0.01; 0.01) | (0.01; 0.01) | (0.01; 0.01) | (0.01; 0.01) | (0.01; 0.01) | (0.01; 0.01) | (0.01; 0.01) | (0.01; 0.01) | (0.01; 0.01) |

| | | | | | | | | | |
|---|---|---|---|---|---|---|---|---|---|
| Unmarried | -0.07 | -0.01 | -0.04 | -0.13 | -0.01 | -0.07 | -0.27*** | -0.26*** | -0.26*** |
| | (-0.21; 0.07) | (-0.14; 0.12) | (-0.16; 0.08) | (-0.39; 0.13) | (-0.24; 0.22) | (-0.30; 0.15) | (-0.43; -0.12) | (-0.40; -0.11) | (-0.39; -0.12) |
| Separated | -0.42*** | -0.29*** | -0.32*** | -0.36** | -0.14 | -0.21 | -0.58*** | -0.56*** | -0.55*** |
| | (-0.61; -0.22) | (-0.47; -0.11) | (-0.50; -0.14) | (-0.71; -0.00) | (-0.45; 0.17) | (-0.53; 0.10) | (-0.82; -0.35) | (-0.77; -0.35) | (-0.75; -0.34) |
| Divorced | -0.16* | -0.12 | -0.12 | -0.12 | -0.04 | -0.05 | -0.29*** | -0.25*** | -0.26*** |
| | (-0.34; 0.02) | (-0.28; 0.05) | (-0.28; 0.04) | (-0.45; 0.21) | (-0.35; 0.27) | (-0.34; 0.24) | (-0.48; -0.10) | (-0.43; -0.07) | (-0.44; -0.09) |
| Widow | -0.39*** | -0.27** | -0.30*** | -0.20 | 0.20 | -0.01 | -0.13 | -0.11 | -0.10 |
| | (-0.63; -0.15) | (-0.49; -0.04) | (-0.52; -0.09) | (-0.84; 0.43) | (-0.40; 0.79) | (-0.56; 0.54) | (-0.38; 0.12) | (-0.34; 0.12) | (-0.32; 0.13) |
| Schooling >8 years | -0.03 | -0.00 | -0.01 | -0.15 | -0.06 | -0.07 | 0.18 | 0.14 | 0.13 |
| | (-0.21; 0.16) | (-0.18; 0.17) | (-0.18; 0.15) | (-0.45; 0.15) | (-0.33; 0.21) | (-0.33; 0.18) | (-0.04; 0.40) | (-0.08; 0.36) | (-0.08; 0.34) |
| CCHS #2 | 0.21 | 0.01 | 0.07 | 0.21 | -0.09 | -0.04 | 0.17 | 0.15 | 0.20 |
| | (-0.24; 0.66) | (-0.42; 0.43) | (-0.35; 0.49) | (-0.54; 0.96) | (-0.79; 0.60) | (-0.73; 0.65) | (-0.35; 0.68) | (-0.35; 0.65) | (-0.28; 0.68) |
| CCHS #3 | 0.62 | 0.10 | 0.24 | 0.58 | -0.22 | -0.14 | 0.25 | 0.25 | 0.38 |
| | (-0.70; 1.95) | (-1.17; 1.38) | (-1.00; 1.49) | (-1.59; 2.74) | (-2.29; 1.84) | (-2.17; 1.89) | (-1.27; 1.77) | (-1.23; 1.74) | (-1.05; 1.81) |
| CCHS #4 | 0.14 | -0.70 | -0.49 | -0.34 | -1.54 | -1.45 | -0.24 | -0.31 | -0.09 |
| | (-1.96; 2.25) | (-2.72; 1.32) | (-2.47; 1.48) | (-3.77; 3.10) | (-4.82; 1.75) | (-4.68; 1.78) | (-2.65; 2.16) | (-2.66; 2.03) | (-2.36; 2.17) |
| CCHS #5 | -0.07 | -1.23 | -0.96 | -0.72 | -2.36 | -2.29 | -0.75 | -0.84 | -0.53 |
| | (-3.10; 2.96) | (-4.14; 1.68) | (-3.81; 1.88) | (-5.67; 4.22) | (-7.09; 2.38) | (-6.94; 2.35) | (-4.22; 2.71) | (-4.21; 2.54) | (-3.79; 2.73) |
| Constant | 9.43*** | 8.06*** | 8.51*** | 9.42*** | 7.48*** | 7.64*** | 7.91*** | 7.83*** | 8.38*** |
| | (5.88; 12.97) | (4.66; 11.47) | (5.16; 11.86) | (3.67; 15.16) | (1.97; 12.99) | (2.20; 13.08) | (3.85; 11.97) | (3.87; 11.78) | (4.54; 12.22) |
| Observations | 33,144 | 36,314 | 38,252 | 14,752 | 16,022 | 17,008 | 18,392 | 20,292 | 21,244 |
| R-squared | 0.57 | 0.58 | 0.58 | 0.60 | 0.60 | 0.60 | 0.60 | 0.60 | 0.60 |
| N | 16,127 | 17,241 | 17,753 | 7,405 | 7,863 | 8,160 | 8,722 | 9,378 | 9,593 |

Change in SCORE after a myocardial infarction in a parent in the general population, the Copenhagen City Heart Study, using data from the 2nd(#2), 3rd(#3), 4th(#4), and 5th(#5) examinations. Estimates are by fixed-effects regressions, estimating the effect of paternal, maternal, and parental myocardial infarct on own SCORE risk by sex (women and men) and duration of education (≤8 years and >8 years). The fixed-effect estimator was at participant level across observed follow-up periods between the five examinations of the Copenhagen City Heart study. Individuals older than 75 years are excluded, as older age may overestimate the effects on SCORE due to the exponential nature of the measure. Robust confidence intervals are reported in parenthesis. *p value<0.05, **p value<0.01, and ***p value<0.001. N=number, yr.=years, MI=myocardial infarction, CCHS=Copenhagen City Heart Study.

## Supplementary Table 3 – extension of data shown in Figure 2, lower panel

Interactional effects on change in SCORE between level of education and the mother, father or either parent experiencing a myocardial infarction

| | All | | | Men | | | Women | | |
|---|---|---|---|---|---|---|---|---|---|
| | Father | Mother | Parent | Father | Mother | Parent | Father | Mother | Parent |
| Schooling > 8yr * parent with MI | | | -0.00 | | | -0.07 | | | 0.16 |
| | | | (-0.22; 0.21) | | | (-0.47; 0.34) | | | (-0.05; 0.37) |
| Schooling > 8yr * father with MI | -0.07 | | | -0.07 | | | 0.07 | | |
| | (-0.35; 0.22) | | | (-0.61; 0.47) | | | (-0.20; 0.35) | | |
| Schooling > 8yr * mother with MI | | 0.08 | | | 0.01 | | | 0.16 | |
| | | (-0.22; 0.38) | | | (-0.57; 0.60) | | | (-0.15; 0.47) | |
| Father with MI | -0.07 | | | -0.19 | | | -0.04 | | |
| | (-0.32; 0.18) | | | (-0.65; 0.28) | | | (-0.29; 0.21) | | |
| Parent with MI | | | -0.15 | | | -0.20 | | | -0.16* |
| | | | (-0.34; 0.03) | | | (-0.56; 0.16) | | | (-0.34; 0.01) |
| Mother with MI | | -0.33** | | | -0.33 | | | -0.25* | |
| | | (-0.60; -0.06) | | | (-0.89; 0.22) | | | (-0.52; 0.01) | |
| Age, years | -0.57*** | -0.54*** | -0.56*** | -0.60*** | -0.56*** | -0.57*** | -0.53*** | -0.52*** | -0.54*** |
| | (-0.66; -0.49) | (-0.63; -0.46) | (-0.64; -0.48) | (-0.74; -0.46) | (-0.70; -0.43) | (-0.70; -0.44) | (-0.63; -0.43) | (-0.61; -0.42) | (-0.63; -0.45) |
| Age, squared | 0.01*** | 0.01*** | 0.01*** | 0.01*** | 0.01*** | 0.01*** | 0.01*** | 0.01*** | 0.01*** |
| | (0.01; 0.01) | (0.01; 0.01) | (0.01; 0.01) | (0.01; 0.01) | (0.01; 0.01) | (0.01; 0.01) | (0.01; 0.01) | (0.01; 0.01) | (0.01; 0.01) |
| Schooling > 8 years | -0.03 | -0.02 | -0.02 | -0.17 | -0.07 | -0.09 | 0.17 | 0.12 | 0.08 |
| | (-0.22; 0.16) | (-0.20; 0.16) | (-0.20; 0.15) | (-0.48; 0.13) | (-0.35; 0.21) | (-0.35; 0.17) | (-0.07; 0.40) | (-0.11; 0.35) | (-0.14; 0.31) |
| Unmarried | -0.19*** | -0.13* | -0.15** | -0.19 | -0.05 | -0.12 | -0.43*** | -0.42*** | -0.41*** |

| | | | | | | | | | |
|---|---|---|---|---|---|---|---|---|---|
| | (-0.34; -0.05) | (-0.26; 0.00) | (-0.28; -0.03) | (-0.45; 0.08) | (-0.29; 0.18) | (-0.35; 0.11) | (-0.59; -0.27) | (-0.56; -0.27) | (-0.55; -0.27) |
| Separated | -0.68*** | -0.54*** | -0.57*** | -0.56*** | -0.33** | -0.40** | -0.89*** | -0.85*** | -0.83*** |
| | (-0.88; -0.47) | (-0.73; -0.36) | (-0.75; -0.38) | (-0.92; -0.19) | (-0.66; -0.00) | (-0.73; -0.07) | (-1.13; -0.65) | (-1.07; -0.63) | (-1.04; -0.62) |
| Divorces | -0.30*** | -0.25*** | -0.25*** | -0.18 | -0.09 | -0.11 | -0.48*** | -0.43*** | -0.44*** |
| | (-0.48; -0.11) | (-0.42; -0.08) | (-0.42; -0.08) | (-0.52; 0.16) | (-0.41; 0.23) | (-0.41; 0.19) | (-0.68; -0.29) | (-0.62; -0.25) | (-0.62; -0.26) |
| Widowed | -0.41*** | -0.29** | -0.32*** | -0.15 | 0.24 | 0.05 | -0.20 | -0.18 | -0.16 |
| | (-0.66; -0.17) | (-0.52; -0.07) | (-0.54; -0.10) | (-0.79; 0.50) | (-0.36; 0.84) | (-0.51; 0.61) | (-0.46; 0.05) | (-0.41; 0.06) | (-0.39; 0.07) |
| CCHS #2 | 0.27 | 0.06 | 0.13 | 0.23 | -0.05 | 0.01 | 0.26 | 0.20 | 0.25 |
| | (-0.18; 0.73) | (-0.37; 0.50) | (-0.30; 0.55) | (-0.52; 0.99) | (-0.75; 0.65) | (-0.69; 0.71) | (-0.27; 0.78) | (-0.31; 0.71) | (-0.24; 0.74) |
| CCHS #3 | 0.56 | 0.02 | 0.16 | 0.44 | -0.27 | -0.18 | 0.24 | 0.12 | 0.25 |
| | (-0.80; 1.91) | (-1.28; 1.32) | (-1.11; 1.43) | (-1.76; 2.64) | (-2.37; 1.82) | (-2.24; 1.88) | (-1.33; 1.81) | (-1.41; 1.65) | (-1.22; 1.72) |
| CCHS #4 | -0.06 | -0.92 | -0.70 | -0.60 | -1.65 | -1.53 | -0.40 | -0.66 | -0.43 |
| | (-2.21; 2.09) | (-2.98; 1.14) | (-2.71; 1.31) | (-4.09; 2.89) | (-4.98; 1.69) | (-4.81; 1.75) | (-2.88; 2.08) | (-3.07; 1.75) | (-2.76; 1.90) |
| CCHS #5 | -0.33 | -1.52 | -1.22 | -1.05 | -2.46 | -2.35 | -0.95 | -1.31 | -0.98 |
| | (-3.43; 2.76) | (-4.48; 1.45) | (-4.13; 1.68) | (-6.08; 3.98) | (-7.26; 2.34) | (-7.07; 2.37) | (-4.53; 2.62) | (-4.79; 2.16) | (-4.34; 2.38) |
| Constant | 10.86*** | 9.55*** | 10.09*** | 10.68*** | 9.11*** | 9.35*** | 9.59*** | 9.24*** | 9.95*** |
| | (7.24; 14.49) | (6.08; 13.03) | (6.67; 13.51) | (4.84; 16.52) | (3.51; 14.71) | (3.82; 14.87) | (5.39; 13.79) | (5.17; 13.31) | (5.99; 13.92) |
| Observations | 33,144 | 36,314 | 38,252 | 14,752 | 16,022 | 17,008 | 18,392 | 20,292 | 21,244 |
| R-squared | 0.56 | 0.57 | 0.57 | 0.59 | 0.59 | 0.59 | 0.57 | 0.58 | 0.58 |
| N | 16,127 | 17,241 | 17,753 | 7,405 | 7,863 | 8,160 | 8,722 | 9,378 | 9,593 |

Change in SCORE after a myocardial infarction in a parent in the general population, the Copenhagen City Heart Study, using data from the 2nd(#2), 3rd(#3), 4th(#4), and 5th(#5) examinations. Estimates are by fixed-effects regressions, estimating the effect of paternal, maternal, and parental myocardial infarct on own SCORE risk by sex (women and men) and duration of education (≤8 years and >8 years). The fixed-effect

estimator was at participant level across observed follow-up periods between the five examinations of the Copenhagen City Heart study. Individuals older than 75 years are excluded, as older age may overestimate the effects on SCORE due to the exponential nature of the measure. Robust confidence intervals are reported in parenthesis. *p value<0.05, **p value<0.01, and ***p value<0.001. N=number, yr.=years, MI=myocardial infarction, CCHS=Copenhagen City Heart Study.

## Supplementary Table 4 – extension of data shown in Figure 3

Interactional effects between own level of SCORE and the father, mother, or either parent experiencing a myocardial infarction

| | All | | |
|---|---|---|---|
| | Father | Mother | Parent |
| Own prior score 5-10 * Parent with MI | | | -2.55*** |
| | | | (-4.36; -0.73) |
| Own prior score > 10 * Parent with MI | | | 4.23 |
| | | | (-3.35; 11.81) |
| Own prior score 5-10 * Father with MI | -3.73*** | | |
| | (-5.85; -1.62) | | |
| Own prior score > 10 * Father with MI | 5.84** | | |
| | (0.89; 10.79) | | |
| Own prior score 5-10 * Mother with MI | | -0.67 | |
| | | (-3.47; 2.12) | |
| Own prior score > 10 * Mother with MI | | 2.73 | |
| | | (-9.21; 14.66) | |
| Parent with MI | | | -0.11 |
| | | | (-0.28; 0.07) |
| Father with MI | -0.12 | | |
| | (-0.35; 0.11) | | |
| Mother with MI | | -0.28** | |
| | | (-0.53; -0.03) | |
| Own prior score 5-10 | 4.53*** | 3.79*** | 4.24*** |
| | (3.30; 5.76) | (2.73; 4.85) | (3.11; 5.37) |
| Own prior score > 10 | -0.10 | -0.14 | -0.19 |
| | (-4.55; 4.35) | (-5.24; 4.96) | (-4.67; 4.28) |
| Unmarried | 1.27* | 1.45** | 1.42** |
| | (-0.06; 2.61) | (0.19; 2.72) | (0.22; 2.63) |
| Separated | 1.01 | 1.17* | 1.17* |
| | (-0.32; 2.34) | (-0.10; 2.43) | (-0.03; 2.36) |
| Divorced | 1.35** | 1.53** | 1.47** |

| | | | |
|---|---|---|---|
| | (0.01; 2.69) | (0.25; 2.80) | (0.26; 2.68) |
| Widow | 1.26* | 1.42** | 1.39** |
| | (-0.10; 2.62) | (0.13; 2.71) | (0.17; 2.62) |
| Schooling > 8 years | -0.11 | 0.00 | -0.03 |
| | (-0.43; 0.20) | (-0.31; 0.31) | (-0.31; 0.26) |
| CCHS #3 | 0.70 | 1.04 | 1.01 |
| | (-0.77; 2.16) | (-0.40; 2.47) | (-0.36; 2.37) |
| CCHS #4 | 0.27 | 0.86 | 0.80 |
| | (-2.47; 3.01) | (-1.82; 3.53) | (-1.75; 3.34) |
| CCHS #5 | 0.12 | 1.04 | 0.93 |
| | (-4.14; 4.39) | (-3.13; 5.20) | (-3.03; 4.89) |
| Age, years | -0.77*** | -0.81*** | -0.81*** |
| | (-0.91; -0.63) | (-0.94; -0.67) | (-0.94; -0.68) |
| Age, squared | 0.01*** | 0.01*** | 0.01*** |
| | (0.01; 0.01) | (0.01; 0.01) | (0.01; 0.01) |
| Constant | 15.47*** | 16.91*** | 16.86*** |
| | (8.91; 22.03) | (10.51; 23.31) | (10.75; 22.98) |
| Observations | 17,475 | 19,187 | 20,129 |
| R-squared | 0.63 | 0.63 | 0.63 |
| N | 10,912 | 11,805 | 12,191 |

Change in SCORE after a myocardial infarction in a parent and stratified by own SCORE prior to parental event in the general population, the Copenhagen City Heart Study, using data from the $2^{nd}$(#2), $3^{rd}$(#3), $4^{th}$(#4), and $5^{th}$(#5) examinations. Estimates are by fixed-effects regressions, estimating the effect of paternal, maternal, and parental myocardial infarct on own SCORE risk by own SCORE categorized as <5%, 5-9.9%, and ≥10% fatal 10-years cardiovascular disease risk. The fixed-effect estimator was at participant level across observed follow-up periods between examination 2-5 of the Copenhagen City Heart study. Individuals older than 75 years are excluded, as older age may overestimate the effects on SCORE due to the exponential nature of the measure. Robust confidence intervals are reported in parenthesis. *p value<0.05, **p value<0.01, and ***p value<0.001. N=number, yr.=years, MI=myocardial infarction, CCHS=Copenhagen City Heart Study.

## Supplementary Table 5 – extension of data shown in Figure 4

Change in own risk factors following a myocardial infarct in the father, mother or either parent stratified by baseline risk

| | Smoker, non-smoker/smoker | | | Systolic blood pressure mmHg | | | Total cholesterol, mmol/L | | |
|---|---|---|---|---|---|---|---|---|---|
| | Father | Mother | Parent | Father | Mother | Parent | Father | Mother | Parent |
| Parent with MI | -0.00 | | | -0.75 | | | -0.03 | | |
| | (-0.03 - 0.03) | | | (-2.43 - 0.92) | | | (-0.12 - 0.07) | | |
| Own prior score 5-10 * Parent with MI | -0.17 | | | 3.35 | | | 0.10 | | |
| | (-0.38 - 0.05) | | | (-6.72 - 13.43) | | | (-0.43 - 0.63) | | |
| Own prior score > 10 * Parent with MI | 0.21* | | | 8.86 | | | 0.30 | | |
| | (-0.02 - 0.43) | | | (-39.33 - 57.05) | | | (-0.98 - 1.57) | | |
| Father with MI | | -0.02 | | | 1.08 | | | -0.06 | |
| | | (-0.05 - 0.01) | | | (-0.59 - 2.75) | | | (-0.15 - 0.04) | |
| Own prior score 5-10 * Father with MI | | -0.01 | | | -14.69*** | | | -0.08 | |
| | | (-0.17 - 0.14) | | | (-23.77 - - 5.61) | | | (-0.58 - 0.41) | |
| Own prior score > 10 * Father with MI | | 0.25** | | | -1.74 | | | 1.04* | |
| | | (0.04 - 0.46) | | | (-26.15 - 22.67) | | | (-0.19 - 2.27) | |
| Mother with MI | | | -0.00 | | | -1.02 | | | -0.05 |
| | | | (-0.04 - 0.03) | | | (-2.76 - 0.72) | | | (-0.15 - 0.05) |
| Own prior score 5-10 * Mother with MI | | | -0.17 | | | 3.88 | | | 0.12 |

| | | | | | | | | | |
|---|---|---|---|---|---|---|---|---|---|
| | | | (-0.39 - 0.05) | | | (-6.56 - 14.31) | | | (-0.44 - 0.67) |
| Own prior score > 10 * Mother with MI | | | 0.20* | | | 8.62 | | | 0.33 |
| | | | (-0.03 - 0.42) | | | (-39.52 - 56.75) | | | (-0.99 - 1.64) |
| Own prior score 5-10 | -0.08*** | -0.05 | -0.09*** | -9.49*** | -7.75*** | -9.43*** | -0.52*** | -0.56*** | -0.53*** |
| | (-0.15 - -0.02) | (-0.12 - 0.02) | (-0.16 - -0.03) | (-12.71 - - 6.26) | (-11.45 - - 4.05) | (-12.76 - - 6.10) | (-0.70 - - 0.33) | (-0.78 - - 0.34) | (-0.73 - - 0.33) |
| Own prior score > 10 | -0.10 | -0.14 | -0.09 | -24.29*** | -23.28*** | -24.05*** | -1.15* | -1.15** | -1.17* |
| | (-0.31 - 0.11) | (-0.34 - 0.07) | (-0.30 - 0.12) | (-33.04 - - 15.55) | (-34.11 - - 12.46) | (-33.11 - - 14.99) | (-2.41 - 0.12) | (-2.16 - - 0.14) | (-2.47 - 0.13) |
| Unmarried | 0.02 | -0.02 | 0.02 | 5.52 | 6.67 | 5.70 | 0.46 | 0.26 | 0.47 |
| | (-0.09 - 0.12) | (-0.07 - 0.03) | (-0.09 - 0.13) | (-3.30 - 14.33) | (-2.31 - 15.65) | (-3.58 - 14.98) | (-0.20 - 1.12) | (-0.16 - 0.69) | (-0.23 - 1.16) |
| Separated | 0.02 | -0.03 | 0.02 | 7.30 | 8.48* | 7.59 | 0.46 | 0.26 | 0.46 |
| | (-0.09 - 0.12) | (-0.09 - 0.03) | (-0.09 - 0.13) | (-1.63 - 16.22) | (-0.62 - 17.58) | (-1.83 - 17.01) | (-0.20 - 1.12) | (-0.17 - 0.68) | (-0.23 - 1.15) |
| Divorced | 0.05 | -0.01 | 0.06 | 4.97 | 6.82 | 5.07 | 0.32 | 0.16 | 0.31 |
| | (-0.06 - 0.15) | (-0.06 - 0.04) | (-0.05 - 0.17) | (-3.88 - 13.82) | (-2.19 - 15.83) | (-4.24 - 14.38) | (-0.34 - 0.99) | (-0.27 - 0.59) | (-0.38 - 1.01) |
| Widow | 0.04 | 0.00 | 0.04 | 5.23 | 6.86 | 5.56 | 0.52 | 0.30 | 0.52 |
| | (-0.07 - 0.14) | (-0.05 - 0.06) | (-0.07 - 0.15) | (-3.70 - 14.17) | (-2.28 - 15.99) | (-3.83 - 14.96) | (-0.15 - 1.18) | (-0.13 - 0.73) | (-0.18 - 1.22) |
| Schooling > 8 years | -0.00 | -0.00 | 0.00 | -0.20 | -1.10 | -0.32 | -0.02 | -0.03 | -0.02 |
| | (-0.03 - 0.03) | (-0.04 - 0.03) | (-0.03 - 0.04) | (-2.07 - 1.68) | (-3.19 - 0.99) | (-2.25 - 1.60) | (-0.11 - 0.07) | (-0.14 - 0.07) | (-0.11 - 0.08) |
| CCHS #3 | -0.00 | -0.05 | 0.02 | 14.65*** | 15.76*** | 13.67*** | 1.15*** | 1.00*** | 1.09*** |
| | (-0.16 - 0.16) | (-0.22 - 0.12) | (-0.14 - 0.18) | (6.45 - 22.84) | (6.92 - 24.60) | (5.29 - 22.05) | (0.66 - 1.63) | (0.48 - 1.53) | (0.59 - 1.59) |
| CCHS #4 | -0.03 | -0.12 | -0.00 | 29.00*** | 30.65*** | 27.23*** | 1.22*** | 0.99** | 1.12** |
| | (-0.33 - 0.26) | (-0.43 - 0.20) | (-0.30 - 0.30) | (13.75 - 44.26) | (14.19 - 47.10) | (11.63 - 42.83) | (0.31 - 2.13) | (0.02 - 1.96) | (0.18 - 2.06) |
| CCHS #5 | -0.06 | -0.20 | -0.01 | 47.76*** | 50.48*** | 45.14*** | 2.06*** | 1.68** | 1.90** |
| | (-0.51 - 0.40) | (-0.69 - 0.29) | (-0.47 - 0.45) | (24.03 - 71.50) | (24.86 - 76.10) | (20.86 - 69.41) | (0.64 - 3.47) | (0.17 - 3.18) | (0.44 - 3.36) |
| Age, years | -0.01 | -0.00 | -0.01 | -1.18*** | -1.34*** | -1.09*** | 0.06** | 0.07*** | 0.06** |
| | (-0.02 - 0.01) | (-0.02 - 0.02) | (-0.02 - 0.01) | (-1.96 - -0.40) | (-2.19 - -0.50) | (-1.89 - -0.29) | (0.01 - 0.10) | (0.02 - 0.11) | (0.01 - 0.11) |
| Age, squared | -0.00 | -0.00 | -0.00 | 0.00*** | 0.01*** | 0.00*** | -0.00*** | -0.00*** | -0.00*** |

| | | | | | | | | | |
|---|---|---|---|---|---|---|---|---|---|
| | (-0.00 - 0.00) | (-0.00 - 0.00) | (-0.00 - 0.00) | (0.00 - 0.01) | (0.00 - 0.01) | (0.00 - 0.01) | (-0.00 - - 0.00) | (-0.00 - - 0.00) | (-0.00 - - 0.00) |
| Constant | 0.85** | 0.63* | 0.92** | 172.54*** | 177.35*** | 168.15*** | 4.88*** | 4.56*** | 4.64*** |
| | (0.15 - 1.54) | (-0.11 - 1.37) | (0.21 - 1.62) | (135.12 - 209.96) | (137.17 - 217.53) | (129.95 - 206.35) | (2.65 - 7.10) | (2.24 - 6.87) | (2.35 - 6.94) |
| Observations | 20,255 | 17,812 | 19,559 | 20,141 | 17,712 | 19,451 | 19,922 | 17,526 | 19,24 |
| R-squared | 0.11 | 0.10 | 0.11 | 0.26 | 0.27 | 0.26 | 0.18 | 0.18 | 0.18 |
| N | 12,295 | 11,073 | 11,981 | 12,254 | 11,037 | 11,943 | 12,136 | 10,933 | 11,828 |

Change in own risk factors after a myocardial infarction in a parent in the general population, the Copenhagen City Heart Study, using data from the 2$^{nd}$(#2), 3$^{rd}$(#3), 4$^{th}$(#4), and 5$^{th}$(#5) examinations. Estimates are by fixed-effects regressions, estimating the effect of paternal, maternal, and parental myocardial infarct on own risk factors (smoking, body mass index, total cholesterol, physical activity, systolic blood pressure, and lipid lowering treatment). The fixed-effect estimator was at participant level across observed follow-up periods between the five examinations of the Copenhagen City Heart study. Individuals older than 75 years are excluded. Robust confidence intervals are reported in parenthesis. *p value<0.05, **p value<0.01, and ***p value<0.001. N=number, yr.=years, MI=myocardial infarction, CCHS=Copenhagen City Heart Study
